# Simulation of Disturbance Recovery Based on MPC and Whole-Body Dynamics Control of Biped Walking

**DOI:** 10.3390/s20102971

**Published:** 2020-05-24

**Authors:** Xuanyang Shi, Junyao Gao, Yizhou Lu, Dingkui Tian, Yi Liu

**Affiliations:** 1 School of Mechatronical Engineering, Intelligent Robotics Institute, Beijing Institute of Technology, Beijing 100081, China; shixuanyang@bit.edu.cn (X.S.); 3120180169@bit.edu.cn (Y.L.); tiandingkui@bit.edu.cn (D.T.); 2 Beijing Advanced Innovation Center for Intelligent Robots and Systems, Beijing 100081, China; 3 Beijing Institute of Astronautical Systems Engineering, Beijing 100076, China; yiliu@bit.edu.cn

**Keywords:** MPC, whole-body dynamics, quadratic optimization, disturbance recovery

## Abstract

Biped robots are similar to human beings and have broad application prospects in the fields of family service, disaster rescue and military affairs. However, simplified models and fixed center of mass (COM) used in previous research ignore the large-scale stability control ability implied by whole-body motion. The present paper proposed a two-level controller based on a simplified model and whole-body dynamics. In high level, a model predictive control (MPC) controller is implemented to improve zero moment point (ZMP) control performance. In low level, a quadratic programming optimization method is adopted to realize trajectory tracking and stabilization with friction and joint constraints. The simulation shows that a 12-degree-of-freedom force-controlled biped robot model, adopting the method proposed in this paper, can recover from a 40 Nm disturbance when walking at 1.44 km/h without adjusting the foot placement, and can walk on an unknown 4 cm high stairs and a rotating slope with a maximum inclination of 10°. The method is also adopted to realize fast walking up to 6 km/h.

## 1. Introduction

Humanoid robots imitate the form of human biped walking with the aim to move in unstructured complex environments and overcome external disturbances [1,2]. Compared with wheeled and tracked robots, humanoid robots have a stronger adaptability in complex environments and a wide range of applications in many fields. A good performance for a walking robot can be simply defined as moving from a starting to a goal point without falling down within disturbance, which is usually caused by external force disturbance, uneven ground and unmodeled dynamics in high dynamic motion. In this sense, the main objective of the control in a walking robot is to guarantee zero moment point (ZMP) and body posture to keep the dynamic balance [3]. However, generating fast and stable walking under disturbance for humanoid robots is a multidisciplinary and complex subject, due to the naturally unstable dynamics of these types of robots. To reduce the difficulty of robot stability control, the overall structure of the walking system in preview works is usually decoupled into four hierarchy levels, which are footstep planner, reference generators, disturbance recovery and low-level controller [4]. The preview methods usually use preview control or differential dynamic program (DDP) to plan the offline trajectory, fix the center of mass (COM) to the waist, and use linear quadratic regulator (LQR) [5] controllers based on the linear inverted pendulum model (LIPM) to keep the robot’s stability by keeping the ZMP inside the support polygon. The method controls the ZMP by adding a small center of mass (COM) trajectory adjustment amount. When the disturbance is too large, the control is easy to diverge and it is difficult to realize large disturbance recovery. However, the COM is not fixed to a point on the body during the robot’s motion, but rather changes dynamically. Otherwise, the authors of this paper believe that stable walking can be realized by controlling whole-body instantaneous dynamics to ensure that ZMP at each control period is in the support area instead of perfect tracking.

In recent years, many authors have studied the subject of humanoid balance control. The key issue in the matter is to look for a fast algorithm that allows a control even if the robot is subjected to impulsive disturbances caused by external force or unknown uneven ground. Furusho [6] stabilize whole-body balance or posture by using the ankle torque of the support leg by modeling the whole robot as a simple inverted pendulum. Nagasaka introduced a torso position compliance (TPC) control method [7]. This control law is effective for a walking robot with feet of high stiffness and was used to stabilize humanoid robots H5 and H7 [8]. Hirai et al. proposed the model ZMP control method [9]. The main idea is when the body of the real robot is inclined more forward than the model, the model body is more strongly accelerated than the planned trajectory. This changes the target inertial force and the target ZMP then goes more backward than the original ZMP, hence producing posture recovery in the real robot. Yu et al. proposed a body acceleration and foot placement adjustment method to reject disturbance [10]. Li et al. proposed a novel control framework to demonstrate a unique foot tilting maneuver based on ankle torque control for humanoid balance recovery [11]. These methods are only based on the simplified model or certain parts and they are all based on the current optimality; they have achieved good results but are difficult to adapt to large disturbances.

When a human is in motion, most joints of the body participate in the movement to maintain balance within disturbance [12]. Using the whole body to maintain balance is better than the simplified model. Yoshino [13] model the walking robot as a multi-input-multi-output (MIMO) system having joint torques as its input vector and output as the state of all links. By applying a LQR controller, the robot could successfully walk at 3 km/h on the floor with an unevenness of 6 mm. This method needs to linearize the robot system, which will lose more characteristics of the robot. Khatib [14] first used inverse dynamics to map the task space control to the joint space control, calculating the joint acceleration or joint moment; this allows us to exert control at the task space and map to the joint space, indirectly achieving nonlinear control. This method relies on convex optimization to solve tracking and constraint problems. Hutter et al. treated the floating inverse dynamics problem as a quadratic programming (QP) problem and provided a method of solving inverse dynamics problems with constraints [15]. Stephane Caron et al. extend walking stabilization based on LIPM tracking by quadratic programming-based wrench distribution, and they showed that the HRP-4 was able to climb an industrial staircase with 18.5 cm-high steps [16]. Feng et al. applied the optimization-based inverse kinematic and inverse dynamic control method in atlas at the defense advanced research projects agency (DARPA) challenge [17]. However, the instantaneous stability is achieved only by whole-body control, and the pre-planned trajectory is not adjusted in real-time in the case of large-scale disturbances.

**Figure 1:**
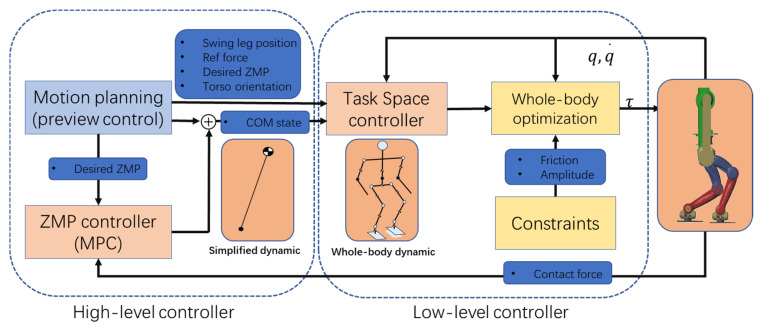
Block diagram of whole-body motion control. q and $\overset{\cdot }{q}$ are joints position and velocity, and τ is the joints torque.

In this paper, a two-level controller of the simplified model and the whole-body model to reject disturbance, instead of using the simplified model and fixed COM, is proposed; the whole control is divided into two levels, the high level is motion planning control based on the LIP model, the low level is task space controller, including trajectory tracking, environment constraints and whole-body ZMP control based on whole-body dynamics (as shown in Figure 1). Firstly, we use the preview control method to generate the walking pattern. The motion planning section also generates reference arm motion trajectories and reference contact forces. In addition, we design a real-time MPC controller to stabilize ZMP. Secondly, a feedback and feedforward controller are applied to calculate the task space acceleration. Thirdly, a whole-body dynamics controller is implemented considering the friction constraint and joint constraint. The QP optimization method is adopted to map the task space acceleration and expected ZMP to joint torque. Finally, a physical simulation environment is established to verify the proposed method and to test external force disturbance, walking on uneven ground and fast walking.

There are three main contributions of this paper. The first is using MPC controller to stabilize ZMP to get future optimization, instead of LQR that considers current optimization. The second is using whole-body dynamics, which is more like the real robot dynamics, to get a wider range of disturbance rejection abilities. The third is that we considered the environment constraint to get better environmental adaptability by using QP optimization.

## 2. High-Level Trajectory Planning and Control

### 2.1. Motion Planning

This section mainly introduces the motion planning method, using the preview control method proposed by Kajita [5] to generate the walking pattern. The main principle is simplifying the robot as a linear inverted pendulum, fixing the vertical height of the COM and using the future foot placement information to plan the COM motion in advance, which is similar to model predictive control. Given the predefined foot placements with a gait cycle and step length, the desired ZMP is located in the support foot in the single support phase and moves to the next support foot in the double support phase by a cubic interpolation curve. Then, given the COM height, the contact force and COM trajectory, including position and velocity, can be calculated.

**Figure 2:**
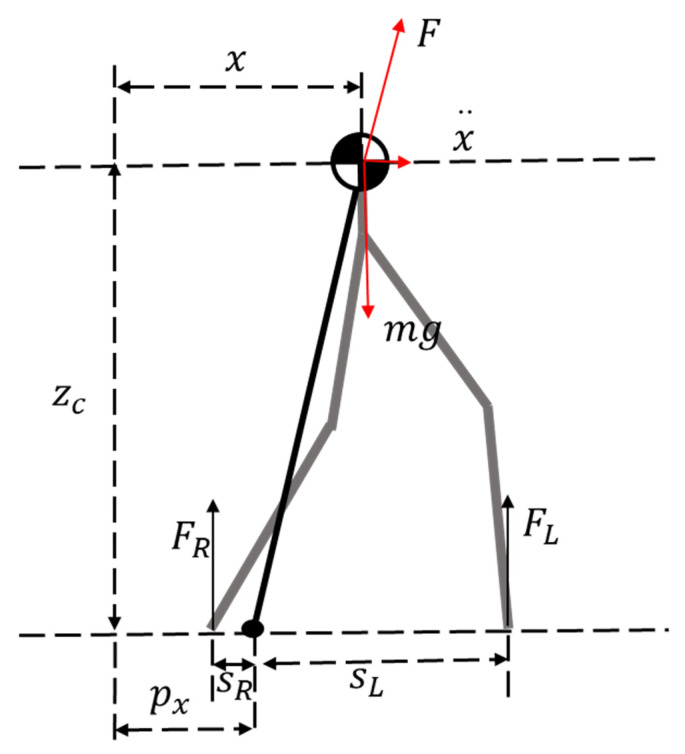
The linear inverted pendulum model of the humanoid robot.

Figure 2 shows the linear inverted pendulum model of the humanoid robot. Equations (1) and (2) are the dynamical system of 3D LIPM. State x is the COM position, u is the control input, $z_{c}$ is the COM height, $p_{x}$ is ZMP and $X={\left[\begin{array}{ccc} x & \overset{\cdot }{x} & \overset{\cdot \cdot }{x} \end{array}\right]}^{T}$ is the state vector.

(1) $$\frac{d}{dt}\left[\begin{array}{c} x\\ \overset{\cdot }{x}\\ \ddot{x} \end{array}\right]=\left[\begin{array}{ccc} 0 & 1 & 0\\ 0 & 0 & 1\\ 0 & 0 & 0 \end{array}\right]\left[\begin{array}{c} x\\ \overset{\cdot }{x}\\ \ddot{x} \end{array}\right]+\left[\begin{array}{c} 0\\ 0\\ 1 \end{array}\right]u$$

(2) $$p_{x}=\left[\begin{array}{ccc} 1 & 0 & z_{c}/g \end{array}\right]\left[\begin{array}{c} x\\ \overset{\cdot }{x}\\ \overset{\cdot \cdot }{x} \end{array}\right]$$

The cost function J of the preview controller and control input u(k) of walking is:

(3) $$J=\overset{\infty }{\underset{i=k}{\sum }}\left\{Q_{e}e{\left(i\right)}^{2}+\Delta X^{T}\left(i\right)Q_{X}\Delta X\left(i\right)+R_{X}\Delta u{\left(i\right)}^{2}\right\}$$

(4) $$u\left(k\right)=-G_{i}\overset{k}{\underset{i=0}{\sum }}e\left(k\right)-G_{x}X\left(k\right)-\overset{N_{L}}{\underset{j=1}{\sum }}G_{p}\left(j\right)p^{ref}\left(k+j\right)$$

where $e\left(i\right)=p\left(i\right)-p^{ref}\left(i\right)$ is the ZMP error, ΔX(i) is the state change increment, Δu(i) is the control input, $G_{i}$ is the ZMP feedback gain, $G_{X}$ is the state feedback gain and $G_{p}\left(j\right)$ is the future ZMP feedforward gain. $Q_{e}$, $Q_{X}$ and $R_{X}$ are the weight matrix. The control input contains not only the feedback of the current state and the current ZMP error but also reference ZMP information for $N_{L}$ future control cycles. According to the reference ZMP information for the future, the controller can predict the future COM trajectory in advance, so that the COM motion velocity and acceleration will not be too large when the ZMP changes. The preview control weights are $Q_{e}=1$, $R_{x}=10e-8$, $Q_{x}=0$ and $N_{L}=500$. The control gains can be calculated off-line by using MATLAB function dare to solve the Riccati equation. A sixth-order curve interpolation is used to generate the trajectory of the swinging leg.

In the single support phase, the contact force of the support foot in the z direction is mg, the swing foot is 0. In the double support phase, the contact force can be calculated by the foot position and ZMP. The contact forces satisfy the following relationships as:

(5) $$\{\begin{array}{c} F_{R}=\frac{s_{L}}{s_{R}+s_{L}}mg\\ F_{L}=\frac{s_{R}}{s_{R}+s_{L}}mg \end{array},$$

Adopting this preview control method, the COM trajectory, foot trajectory and reference contact force are obtained.

### 2.2. Model Predictive Control of ZMP

Thinking about a stabilization method based on a LIP model. In this case we must measure the ZMP to design a feedback controller. As mentioned in [7,8], previous researchers used LQR to control the acceleration of the center of mass to stabilize ZMP. However, LQR only pursues the current optimal, resulting in a relatively large control input. Thus, an MPC controller is implemented in this paper to improve the control performance.

The ZMP equation calculated by the LIP model is:

(6) $${}^{B}p_{x,cal}=x-\frac{z_{c}}{g}\ddot{x},$$

Define the measured ZMP as ${}^{B}p_{x,rel}$, and, by assuming the time constant of the ZMP sensor as T, the relationship between measured ZMP and calculated ZMP is:

(7) $${}^{B}p_{x,rel}=\frac{1}{1+Ts}{}^{B}p_{x,cal},$$

Rewrite this as an error state function as (8), $\Delta X_{c}={\left[{}^{B}\Delta p_{x,rel},\Delta x,\Delta \overset{\cdot }{x}\right]}^{T}$ is the error state and $u_{c}=\ddot{\Delta x}$ is the control input. It is noteworthy that the control quantity is an additional acceleration on the original trajectory.

(8) $$\frac{d}{dt}\left[\begin{array}{c} {}^{B}\Delta p_{x,rel}\\ \Delta x\\ \Delta \overset{\cdot }{x} \end{array}\right]=\left[\begin{array}{ccc} -\frac{1}{T} & \frac{1}{T} & 0\\ 0 & 0 & 1\\ 0 & 0 & 0 \end{array}\right]\left[\begin{array}{c} {}^{B}\Delta p_{x,rel}\\ \Delta x\\ \Delta \overset{\cdot }{x} \end{array}\right]+\left[\begin{array}{c} -\frac{z_{c}}{gT}\\ 0\\ 1 \end{array}\right]\Delta \ddot{x}\;,\;{}^{B}\Delta p_{x,rel}=\left[\begin{array}{ccc} 1 & 0 & 0 \end{array}\right]\left[\begin{array}{c} {}^{B}\Delta p_{x,rel}\\ \Delta x\\ \Delta \overset{\cdot }{x} \end{array}\right],$$

To compute a feasible control input, we solve them through model predictive control as Equation (9). Here we sample all time-varying quantities at N knot points, with the time durations, h, $A_{c}$ and $B_{c}$ being the state matrix after discretization; $Q_{c}$ and R are the weight coefficients. At time zero, given Δx=0 and $\Delta \overset{\cdot }{x}=0$, and it is calculated by integration of the control input. The ${}^{B}\Delta p_{x,rel}$ is updated in each control cycle based on the desired ZMP and the measured ZMP. Add the values Δx, $\Delta \overset{\cdot }{x}$ and $\Delta \ddot{x}$ onto the previously generated trajectory as inputs to the whole-body controller. The optimization problem can be solved in under 1 ms.

(9) $$\begin{array}{cc} \underset{u_{c}\left(k\right)}{min} & \end{array}\overset{N}{\underset{k=1}{\sum }}\left(\Delta X_{c}^{T}\left(k\right)Q_{c}\Delta X_{c}^{T}\left(k\right)+R\Delta u_{c}{\left(k\right)}^{2}\right)\;\begin{array}{ccc} & & st.\begin{array}{cc} & \Delta X_{c}^{T}\left(k+1\right)= \end{array} \end{array}A_{c}\cdot \Delta X_{c}^{T}\left(k\right)+B_{c}\cdot \Delta u_{c}\left(k\right)$$

**Figure 3:**
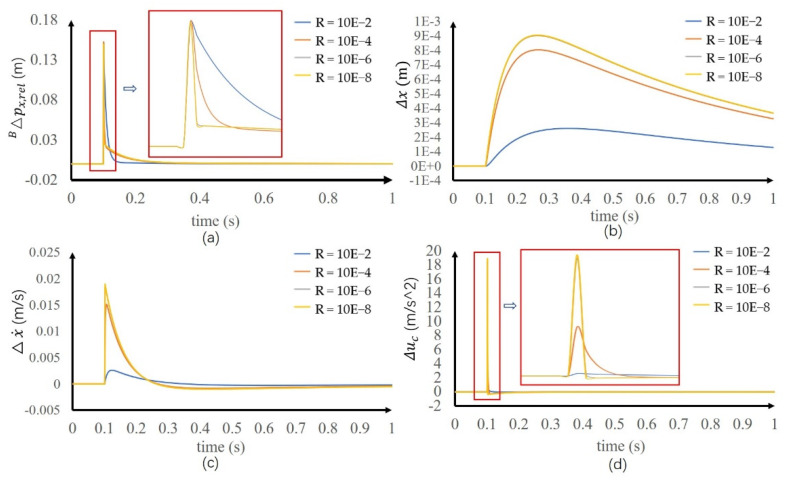
Simulation results of zero moment point (ZMP) error control. (**a**) ZMP error. (**b**) COM position increment. (**c**) COM velocity increment. (**d**) Control input.

The method is numerically simulated to verify the stability. The ParNMPC [18] solver is used to solve this optimization problem. The parameters are N = 12, h = 0.004 s and the control period is 1 ms. The time delay of the force sensor is 50 ms, measured in the previous experiment, and at t = 1 s, a disturbance is applied to the state variable ${}^{B}\Delta p_{x,rel}$ had an amplitude of 0.15 m, which is the maximum ZMP error that can be obtained according to the size of foot. $Q_{c}$ is set to diag([1,1,1]) and R is set to 10^−2^, 10^−4^, 10^−6^ and 10^−8^ to test the performance of different weight coefficient. As shown in Figure 3a. When ZMP error occurs in the system, the controller can quickly eliminate the ZMP error within 0.1 s. Figure 3b,c show that the states Δx and $\Delta \overset{\cdot }{x}$ also go back to equilibrium point 0. By comparing different R parameters, when R is larger, the control quantity u is smaller, resulting in the slow decrease of ZMP error, as shown in Figure 3d. Thus, different control effects can be produced by adjusting R.

## 3. Whole-Body Controller

### 3.1. Modeling

**Figure 4:**
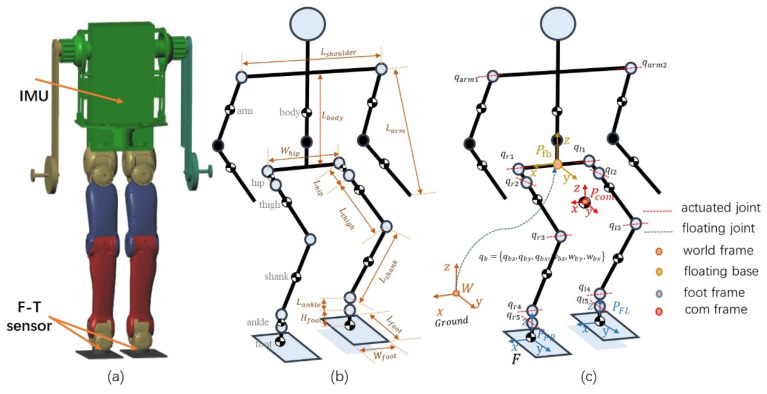
Typical motor-driven humanoid robot. They always have an inertial measurement unit (IMU) fixed inside the chest and a six-dimensional force torque (F-T) sensor for each ankle. Each leg has five degrees of freedom (DoFs): (**a**) Simulation model; (**b**) model mechanical parameters; (**c**) DoFs configuration.

The degrees of freedom (DoFs) layout of the humanoid robot used in this article is shown in Figure 4. There are 18 DoFs in total, including two DoFs on the shoulders, 10 DoFs on the legs and six floating DoFs. The feet of the humanoid robot are not firmly connected to the ground and only provide a supporting force with limited torque. The floating coordinate system is established and fixed to the waist of the robot. $q_{b}=\left\{q_{bz},q_{by},q_{bx},w_{bz},w_{by},w_{bx}\right\}$ is the generalized joint variable of the floating base, which represents the position and Euler angle of the floating base viewed in the world coordinate frame. $q_{r}=\left\{q_{r1},q_{r2},q_{r3},q_{r4},q_{r5}\right\}$ and $q_{l}=\left\{q_{l1},q_{l2},q_{l3},q_{l4},q_{l5}\right\}$ are, respectively, the generalized coordinates of the active joints of the right and left legs. $q_{arm}=\left\{q_{arm1},q_{arm2}\right\}$ denotes the arm joint variables. The robot’s active joints and floating joints are denoted as the robot’s generalized joint coordinate variable $q=\left\{q_{b},q_{r},q_{l},q_{arm}\right\}$, which is an 18 × 1 vector. Each link of the robot has mass and inertia.

A single-support phase, double-support phase and flight phase may appear during robot motion. For convenience of description, dynamics modeling is established for the double-support phase. The single-support phase and flight phase simply require the contact force to be set to zero. The standard form of robotic body dynamics is:

(10) $$D\left(q\right)\overset{\cdot \cdot }{q}+C\left(q,\overset{\cdot }{q}\right)\overset{\cdot }{q}+G\left(q\right)=B\tau +J^{T}\left(q\right)F,$$

To avoid the construction of a contact dynamics model and direct control of the contact force, the dynamic equation is rewritten as:

(11) $$\left[D\left(q\right)-B-J^{T}\left(q\right)\right]\cdot X+C\left(q,\overset{\cdot }{q}\right)\overset{\cdot }{q}+G\left(q\right)=0,$$

In Equation (11), $X={\left[\overset{\cdot \cdot }{q},\tau ,F\right]}^{T}\in {ℜ}^{48\times 1}$ is the generalized state variable of the robot while $\overset{\cdot \cdot }{q}\in {ℜ}^{18\times 1}$ and $\tau \in {ℜ}^{18\times 1}$ are, respectively, the joint acceleration and joint torque. $F\in {ℜ}^{12\times 1}$ is the contact-force variable. The contact-force variable includes two contact points of the left and right feet. Each contact point includes contact forces and contact moments in three directions. $D\left(q\right)\in {ℜ}^{18\times 18}$ is the inertia matrix. $B\in {ℜ}^{18\times 18}$ is the joint torque mapping matrix. $J^{T}\left(q\right)\in {ℜ}^{18\times 12}$ is the Jacobian matrix of the contact point. $C\left(q,\overset{\cdot }{q}\right)\in {ℜ}^{18\times 18}$ is the matrix of the centrifugal force and Coriolis force. $G\left(q\right)\in {ℜ}^{18\times 1}$ is the matrix of the gravity term. In the dynamic equation, the acceleration, torque and contact force are defined as state variables. The advantage is that one or more of the state variables can be directly restricted and controlled. As an example, it is feasible to control the contact force, or switch to the torque output mode or position output mode, which makes the control expression more concise.

The actual COM position of the robot changes during walking. The actual COM position can be calculated in real-time according to the actual joint position and mass parameters of the robot. The COM is calculated as:

(12) $$P_{com}\left(q\right)=\frac{\sum_{i=1}^{n}m_{i}p_{i}\left(q\right)}{\sum_{i=1}^{n}m_{i}},$$

where $m_{i}$ is the mass of link i and $p_{i}$ is the COM position of link i in the world coordinate frame.

During walking, it is necessary to track the position and posture of each foot, the posture of the upper body, the position of each arm and one or more joint positions and perform other tasks. The position in the world coordinate frame is; therefore, eliminated and the relative position between the COM and foot is controlled. All tasks are described by:

(13) $$T\left(q\right)={\left[\begin{array}{ccccc} P_{com\to fr} & P_{com\to fl} & R_{b} & R_{fr} & R_{fl} \end{array}\right]}^{T},$$

In Equation (13), $P_{com\to fr}$ and $P_{com\to fl}$ are the relative positions of the foot and COM in the world coordinate frame, $R_{fr}$ and $R_{fl}$ are the attitude of the foot in the world coordinate frame, $R_{b}$ is the posture of the upper body in the world coordinate frame, and the posture is expressed by the roll, pitch and yaw.

### 3.2. Task Space Controller

In the task space, we only care about the kinematics part of the task space performance, ignoring the dynamics of the robot. A feedforward and a proportional and derivative feedback controller are applied to control the task acceleration. Through configuration of the gains $k_{d}$ and $k_{p}$, the error state can approach the zero equilibrium point. Although different control targets are unified as a task in the task space, the weight of each control task depends on the scenario. In a walking scenario, the tracking of leg dynamics should be good, whereas the error in tracking the upper body and arms only needs to be not too large. When performing working tasks, the body should be flexible and the arm tracking is expected to be good. Setting different parameters for different application scenarios achieves different control effects. Equation (14) is the feedback and feedforward controller. $T_{d}$ is the desired position or orientation, $\overset{\cdot }{T_{d}}$ and $\overset{\cdot \cdot }{T_{d}}$ are its desired velocity and acceleration, respectively, as mentioned in Equation (13). $\overset{\cdot \cdot }{T_{d}^{*}}$ is the newly calculated acceleration.

(14) $$\overset{\cdot \cdot }{T_{d}^{*}}=k_{p}\left(T_{d}-T\right)+k_{p}\left(\overset{\cdot }{T_{d}}-\overset{\cdot }{T}\right)+\overset{\cdot \cdot }{T_{d}},$$

In the support phase, to ensure that the foot is in contact with the ground without slippage and rotation, the velocity and acceleration of the contact point in the world coordinate system are theoretically required to be zero. For control stability, the contact constraint here is set as a soft constraint. That is to say, the expected acceleration value ${\overset{\cdot \cdot }{P}}_{c\mathrm{ontact}}^{*}$ of the contact-related quantity in the task space is set to zero, ignoring the velocity:

(15) $${\overset{\cdot \cdot }{P}}_{c\mathrm{ontact}}^{*}=0,$$

We have obtained the control input acceleration in the task space. Taking the time derivative of Equation (13) twice, the kinematic mapping Equation (12), which maps the task space to the joint space, is obtained:

(16) $$J\left(q\right)\cdot \overset{\cdot \cdot }{q}=\overset{\cdot \cdot }{T}\left(q\right)-\overset{\cdot }{J}\left(q\right)\overset{\cdot }{q},$$

where J(q) is the task-space Jacobian matrix.

Using Equations (14)–(16), an optimization is performed to find the state input that satisfies the task-space control acceleration:

(17) $$\underset{\overset{\cdot \cdot }{q},\tau ,F}{min}{\left\|J\left(q_{a}\right)\cdot \overset{\cdot \cdot }{q}+\overset{\cdot }{J}\left(q_{a}\right)\overset{\cdot }{q_{a}}-\overset{\cdot \cdot }{T_{d}^{*}}(q_{a})\right\|}^{2},$$

where $q_{a}$ is the actual joint angle and $\overset{\cdot \cdot }{q}$ is the joint acceleration after optimization.

### 3.3. ZMP and Force Mapping

Given the contact force and torque, the location of the ZMP in the foot coordinate frame is calculated using Equation (18). The force and torque are expressed in the local coordinate frame of the foot as:

(18) $$p=\left[\begin{array}{c} -{}^{b}M_{y}/{}^{b}F_{z}\\ {}^{b}M_{x}/{}^{b}F_{z} \end{array}\right],$$

A contact force term is introduced to the optimization goal to ensure the stability of the ground contact force and the stability of the solver when the robot is disturbed or walking on uneven ground. The control input mapping by optimization is Equation (19). The $F_{ref}$ is the reference force, and the F is the contact force calculated by model dynamics and control input instead of measured by the force sensor. Equation (19) stabilizes ZMP by controlling instantaneous dynamics of the whole body, while Equation (9) stabilizes ZMP by adjusting the COM trajectory. The two controllers are combined to achieve a wider range of stability control.

(19) $$\underset{\overset{\cdot \cdot }{q},\tau ,F}{min}{\left\|p_{x}^{*}\cdot {}^{b}F_{z}+{}^{b}M_{y}\right\|}^{2}+{\left\|p_{y}^{*}\cdot {}^{b}F_{z}-{}^{b}M_{x}\right\|}^{2}+{\left\|F-F_{ref}\right\|}^{2}.$$

### 3.4. Constraints

**Figure 5:**
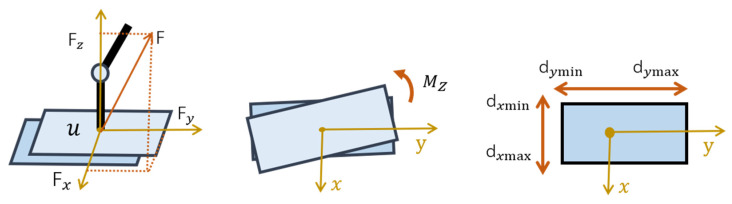
Friction and support region constraints.

When the robot is walking at high speed, the swinging leg rapidly moves in the forward direction, generating a rotational moment in the vertical direction of the supporting foot. This destabilizes the foot–surface contact of the supporting leg and rotates the robot. In addition, motion planning does not consider whether the ground friction meets requirements. Without limits, the robot foot slips on the ground, making the robot unstable, as shown in Figure 5. With our improved dynamic equations, the required frictional force and torque can be adopted as constraints.

The coefficient of ground friction is denoted as u. The contact force needs to meet the conditions given in Equation (20) to prevent slipping. We also added the ZMP constraint to ensure the ZMP is in the support area. The conditions are:

(20) $$\{\begin{array}{c} \left|F_{x}\right|-uF_{z}\leq 0\\ \left|F_{y}\right|-uF_{z}\leq 0\\ \left|M_{z}\right|<M_{max} \end{array}$$

It is necessary to constrain the amplitudes of the control variables in order to avoid joint actuator saturation. Boundary constraints of the acceleration amplitude and moment amplitude can be set according to actual robot hardware conditions:

(21) lb≤X≤ub.

### 3.5. Prioritized Whole-Body Mapping

The task space controller and ZMP controller are often in conflict with each other. We weight the command tasks to select appropriate whole-body joint torques according to task importance through convex optimization. The dynamic equation is used as the constraint equation. The final optimization form is:

(22) $$\begin{array}{l} {\begin{array}{cc} \underset{\overset{\cdot \cdot }{q},\tau ,F}{\min} & \frac{1}{2}w^{2}{}_{task}\left\|J\left(q\right)\overset{\cdot \cdot }{q}+\overset{\cdot }{J}\left(q\right)\overset{\cdot }{q}-\overset{\cdot \cdot }{T_{d}^{*}}(q)\right\| \end{array}}^{2}+\sum \frac{1}{2}w^{2}{}_{zmp}{\left\|p_{x}\cdot {}^{b}F_{z}+{}^{b}M_{y}\right\|}^{2}+\frac{1}{2}w^{2}{}_{F}{\left\|F-F_{ref}\right\|}^{2}\\ s.t.\left[\begin{array}{cc} D\left(q\right) & -B \end{array}-J^{T}\left(q\right)\right]\cdot X+C\left(q,\overset{\cdot }{q}\right)\overset{\cdot }{q}+G\left(q\right)=0\\ d_{x\min}\leq -{}^{b}M_{y}/{}^{b}F_{z}\leq d_{x\max}\\ d_{y\min}\leq {}^{b}M_{x}/{}^{b}F_{z}\leq d_{y\max}\\ \left|F_{x}\right|-uF_{z}\leq 0\\ \left|F_{y}\right|-uF_{z}\leq 0\\ \begin{array}{ccccc} & & & & lb\leq X\leq ub \end{array} \end{array}$$

where $w_{task}=\left[\begin{array}{cc} \begin{array}{cccc} w_{Pcom\to \mathrm{foot}} & w_{Pcom\to foot} & w_{Rb} & w_{\mathrm{foot}} \end{array} & w_{foot} \end{array}\right]$ is the weight coefficient of each task in the task space, $w_{\mathrm{zmp}}$ is the ZMP tracking weight and $w_{F}$ is the contact force weight. Among walking tasks, tracking of the COM trajectory is set to have a high priority and other tasks are set to have a low priority. The general form of quadratic programming is shown as Equation (23), rewrite the general form cost function as $0.5{\left\|A\chi -b\right\|}^{2}$, thus $G=A^{T}A$ and $g=-A^{T}b$. A and b can be decomposed into smaller blocks as Equation (24). Using this rewrite method, the optimization problem (22) can be rewritten into a general form and solved by a standard solver.

(23) minχ0.5χTGχ+gTχst. Aueqχ≤bueq Aeqχ=beq lb≤χ≤ub

(24) $$A=\left[\begin{array}{c} \omega_{0}A_{0}\\ \omega_{1}A_{1}\\ \cdot \\ \omega_{n}A_{n} \end{array}\right],b=\left[\begin{array}{c} \omega_{0}b_{0}\\ \omega_{1}b_{1}\\ \cdot \\ \omega_{n}b_{n} \end{array}\right]$$

The above optimization problem is complex but can be solved by the *MATLAB* QP solver quadprog in 1 ms. The feedback parameters and weight parameters of the method have a significant impact on the results. Although there are many parameters involved in this paper, they all correspond to relatively clear physical meanings. High feedback gain has better tracking effect and low feedback gain has better flexibility. When debugging the parameters, we can first plan a squat down and stand up movement, and set the feedback gain to be larger when moving without disturbance. The weight coefficient is designed according to the priority of task importance to get rough parameters. At high speed, the parameters are adjusted in detail. Generally, the feedback gain is reduced to improve the flexibility. Generally, after one or two days of adjustment the controller has a good performance, and when the dynamic model parameters change, only fine tuning is needed.

## 4. Simulation and Results

**Figure 6:**
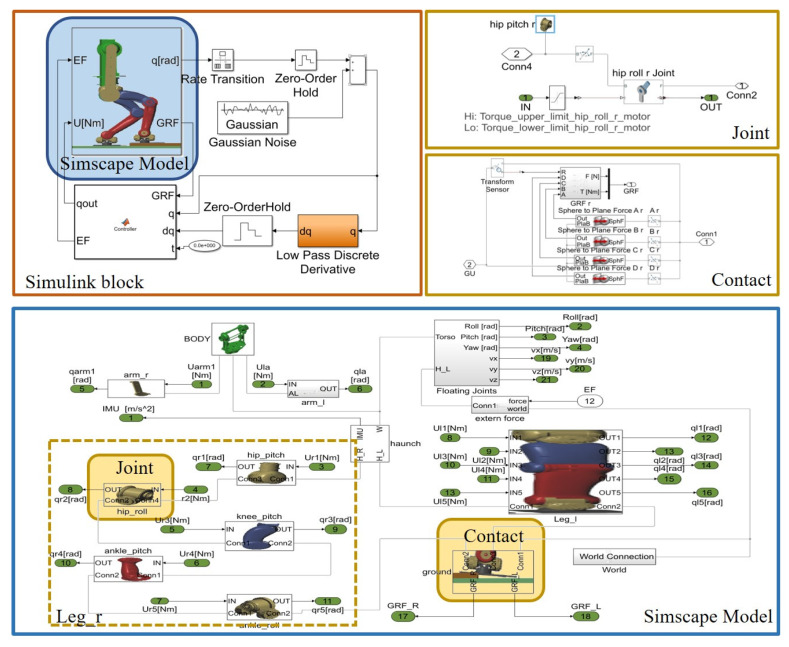
Simscape block of the simulated model.

We used MATLAB2018 and Simscape [19] software to verify the methods mentioned above. Simscape is a toolbox of MATLAB for physics modelling, as a package of Simulink with tools for modelling. As shown in Figure 6, we first designed the mechanical structure of the humanoid robot in SolidWorks and then divided the robot into multiple links with the joint as the segmentation point. In MATLAB the joints are added between the separate links, and the joints take torque as input and angle as output. At the same time, six degrees of freedom free movement joints were added between the body and the world, and their states were measured as inertial measurement unit (IMU) sensor output. For contact, solid-to-solid is not directly used for foot contact. Instead, four balls are added to the four endpoints of the foot to construct a sphere-to-solid contact. The advantage is that the computation is fast and stable, and the SM_Contact_Forces_Lib_R18a_v4p1 contact library is used for contact design in the simulation. To make the simulation more realistic, gaussian noise and low pass filter are added to the state value, including joint velocity, IMU data and contact force.

The simulation is generated in three stages: The first is to verify external force disturbance, second is to verify the unmodeled dynamics disturbance in fast waking and the last is to verify uneven ground disturbance. The total mass of the model is 40.58 kg; the mass of a single leg is about 10.19 kg, the mass of a single arm is 3.1 kg. Table 1 shows the mass and inertia of the links in the model, and also lists the dimensions of the different links of the robotic mechanism. The corresponding variables are labeled in Figure 4b. The coefficient of friction between synthetic rubber and concrete is 0.6–0.85, so the friction coefficient is set to 0.7. The robot has five DOFs on each leg (i.e., two on the hip, one on the knee and two on the ankle) and one DOF on the shoulder in the pitch direction on each arm. Table 2 gives the weights and feedback gains of the task space controller. The weights and feedback gain in Table 1 can be adjusted manually to achieve different control effects.

### 4.1. Disturbance Recovery in Walking

**Figure 7:**
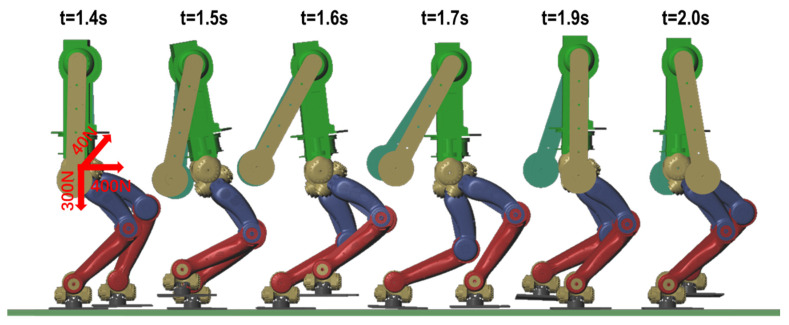
Disturbance recovery in walking.

In verifying the robot’s ability to recover from disturbances in the walking process, we first used a preview control to generate the COM reference trajectory and reference contact force. Given a sequence of 10 foot placements (i.e., 10 steps) with a velocity of 1.44 km/h, the duration of each step is 0.5 s, the step length is 0.2 m, and the period of double support accounts for 5% of the step cycle. As shown in Figure 7, at t = 1.4 s, the left leg of the robot is the supporting leg and the right leg is the swinging leg. An external disturbance is applied to the waist of the robot in the forward, right and vertical directions with magnitudes of 400, 20 and 300 N, respectively. At t = 1.5 s, the disturbance is cancelled (i.e., the disturbance lasts 0.1 s). The robot gains an impulse of 40, 2 and 30 NM in the three directions, respectively. When impacted by external forces, the robot generates additional movement in the direction of the impact to maintain balance. The simulated motion is shown in the supplemental videos (In Supplementary).

**Figure 8:**
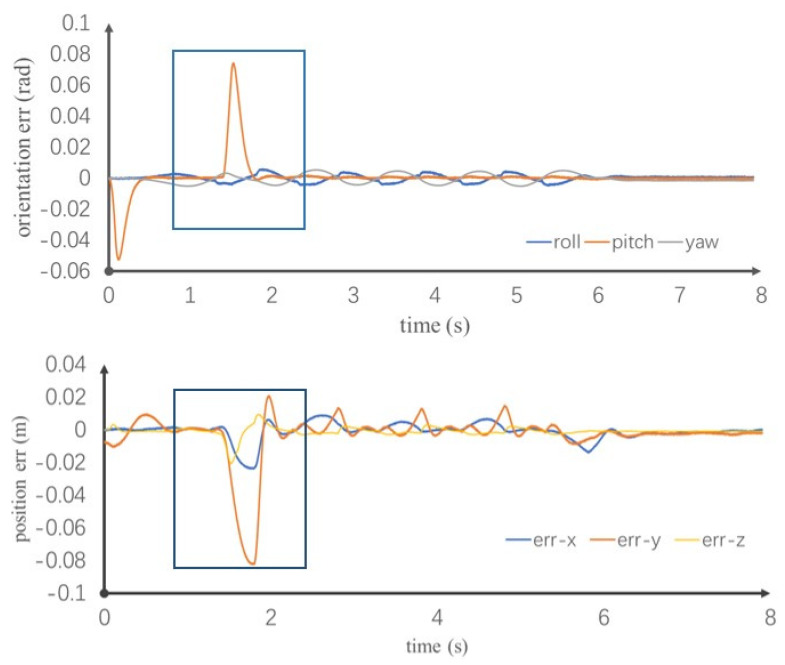
Center of mass (COM) tracking error and body posture error.

Figure 8 shows the COM tracking error and body posture error. At t = 1.4 s, the posture error of the upper body starts to increase. The error reaches a maximum value, about 0.08 rad, at t = 1.52 s and then gradually decreases. The pitch direction returns to the expected value at t = 1.75 s. The posture does not change appreciably in the roll direction upon impact. One reason is that the given impact is small. Another reason is that the speed of the swinging leg in the lateral direction is low during walking. The controller exerts more influence on attitude control. Throughout the walking process, there is a maximum posture error of 0.005 rad in the yaw direction, which is not caused by the rotation or slip of the robot’s foot. This is because the robot model used in this article lacks hip yaw joints, and the pitch joints and roll joints at the hip and ankle are not coplanar. The inverse kinematics are over constrained. It is not possible to guarantee all postures and positions of the foot relative to the body at the same time. Instead, we introduce weights into the calculation. The maximum errors in the location of the COM in the three directions are 0.03, 0.025 and 0.09 m. At t = 1.799 s, the y-direction error reaches a maximum. At t = 2 s, the robot returns to normal walking. When the disturbance disappears, the posture and position error decreases immediately. This is because the speed error generated by the impact is large. To ensure the robot ZMP is located in the foot plate, the controller optimization solution favors stability over COM and posture tracking. During the period t = 2 to 6 s, the robot walks free of disturbance. The error reaches a maximum of 0.02 m. In fact, it is possible to configure a higher gain of the feedback controller to reduce the error, but this is unnecessary because the appropriate gains ensure both tracking and compliance performance.

**Figure 9:**
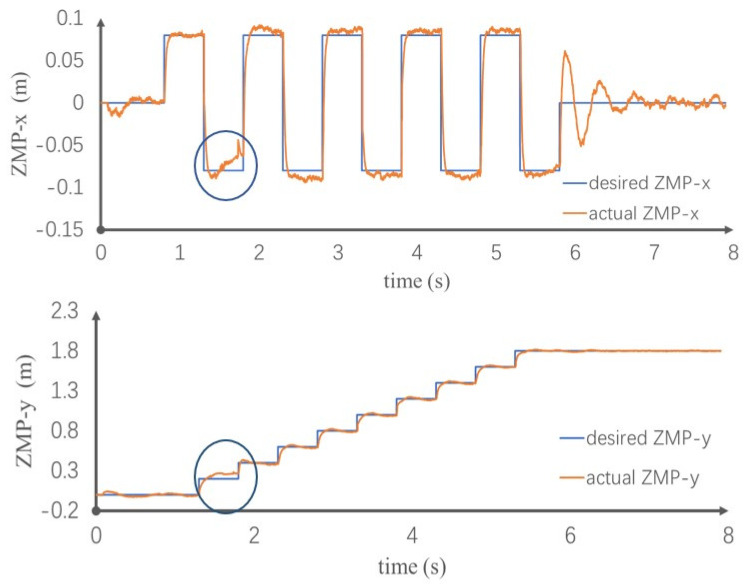
Zero moment point (ZMP) tracking in x and y directions.

Figure 9 shows the ZMP tracking result. When the robot is disturbed by the impact, owing to the compliance provided by the control method, the robot ZMP does not suddenly change, causing the robot’s foot to rotate away from the ground. With the controller, the ZMP slowly changes along the direction of the impact force. When the impact disappears, the track of the actual ZMP gradually approaches the rack of the expected ZMP. Throughout the walking process, the ZMP is located in the stable support area, achieving stable control. The ability to reject 400 N and last 0.1 s external force in this paper is better than literature [20], which is 350 N and 0.05 s, respectively, and it also better than literature [21], which is 6.0 Nm. This simulation proves that when the model is disturbed by external forces, it can quickly recover the original stable walking state and achieve the desired goal.

### 4.2. Fast Walking

**Figure 10:**
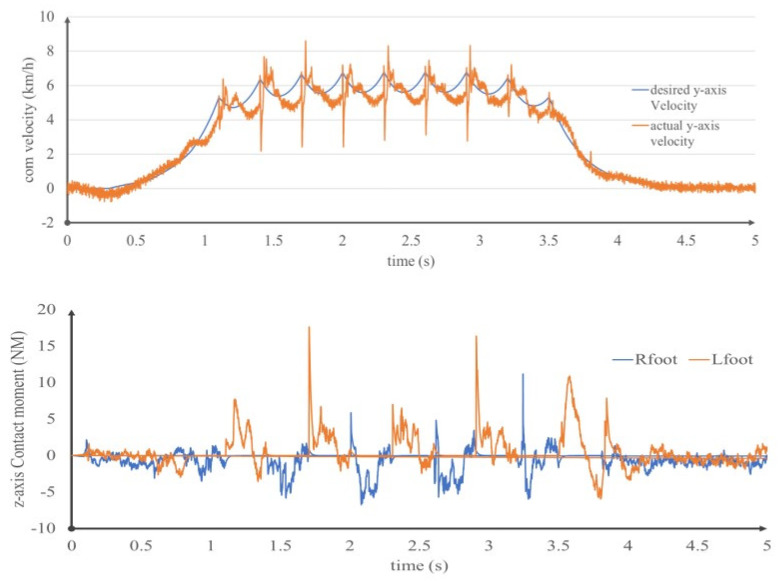
Center of mass (COM) velocity and contact moment in walking at 6 km/h.

We use the proposed controller to achieve fast walking with a velocity of 6 km/h, including acceleration and deceleration gait. Similar to the previous simulation, the step length is set at 0.5 m, the step cycle is set at 0.3 s and there is no period of biped support. Meanwhile, to verify the control performance and model parameters error, we randomly adjust the mass and inertia so that it has an error of plus or minus 15%. Figure 10 shows the COM speed in the forward direction during walking. The robot reaches a maximum speed of 6.2 km/h at t = 1.5 s, which is slightly higher than the intended 6 km/h. The tracking is more accurate when the robot speed is low. During high-speed walking, when the support phase changes, the swinging leg strikes the ground and there is a sudden change in speed. For walking at high speeds, especially with large leg inertia, the rotation torque provided by the motion of the swinging leg is large, which generates a large rotation moment at the support foot. By limiting the contact torque of the foot in this article, the rotation torque of the robot is reduced to 10 Nm. Through this simulation, we prove that the method in this paper can effectively solve the problem of supporting foot rotation caused by the fast swing of the swinging leg when walking at high speed, and control the rotation torque within the limit of friction. In addition, even though the model parameter is not accurate, it can show good robustness.

Fast walking with different friction is also simulated. We changed the set of friction coefficient in the simulation environment and friction constraint of optimization. We tested different friction coefficients with 0.7, 0.4, 0.3, 0.27 and 0.25 in 6 km/h fast walking. Result shows that in 6 km/h walking, the proposed method is able to achieve a minimum friction coefficient of 0.25 walking. The simulated motion is shown in the supplemental video.

### 4.3. Uneven Ground Walking

**Figure 11:**
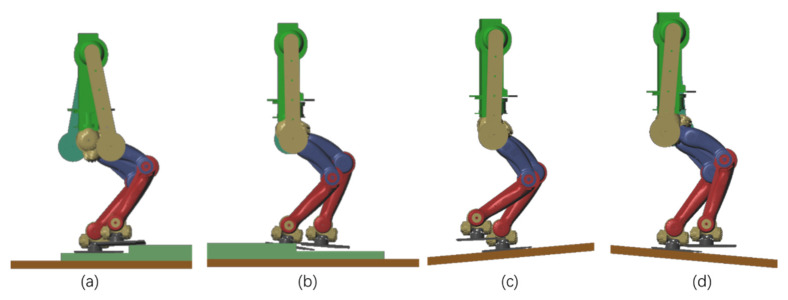
Walking on uneven ground: (**a**) Walking up stairs; (**b**) walking down stairs; (**c**) walking up slope; (**d**) walking down slope.

**Figure 12:**
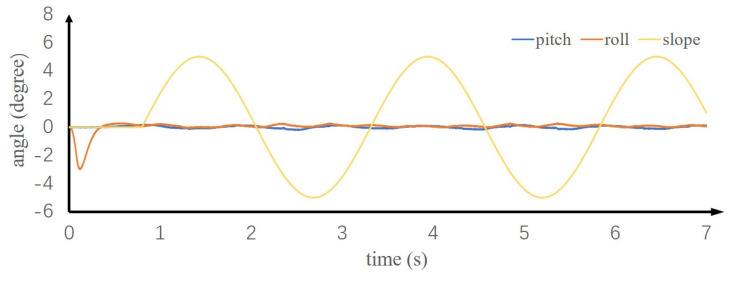
The angle of the body in the direction of pitch and roll while walking on the dynamic ground.

The control method proposed in this paper also has good adaptability for robots walking on unknown uneven ground, which included stairs of unknown size and dynamic slopes of unknown angle. In simulation, the robot does not know where the stairs or slope are, nor does it know the width or height of the stairs and angle of the slope. In order to verify the control performance of the proposed method on the unknown uneven ground, the ground is assumed to be horizontal in motion planning. A 1.44 km/h walking trajectory is planned, and the uneven ground is taken as the disturbance for real-time control. Figure 11a,b show two stairs walking up and two stairs walking down, each stair has a height of 4 cm. Figure 11c,d show the robot walking up and down the slope with a slope angle of 10°. To verify the robot’s ability of walking on dynamic ground, we applied a sine motion with an amplitude of 5° and a period of 2.5 s to the ground. As shown in Figure 12, during the sinusoidal movement of the ground, the measured roll and pitch angles were lower than 0.5°, and the robot was walking steadily. The simulated motion is shown in the supplemental video.

## 5. Discussion

In this paper, the method based on whole-body QP optimization is applied to achieve disturbance recovery. However, it can be predicted that the whole-body optimization using sequential quadratic programming (SQP) or MPC can obtain better control effect, but the calculation cost will be significantly increased, which is difficult to realize under the current calculation conditions. On the other hand, although it is not the research content of this paper, the combination of real-time planning of high-level trajectory based on state feedback, such as recalculating the foot placement and changing the trajectory of the COM, can achieve a larger range of disturbance control. For example, a three-dimensional spring-involved inverted pendulum model and a dead-beat controller could be used to re-plan the trajectory by adjusting the foothold [22], or a real-time gait generation method based on an analytical solution could be used for trajectory adjustment [23]. Despite all above advantages, this controller is implemented only in simulation. Implementing on the experimental setup has more practical challenges. For example, accurate state estimation to obtain the robot state, especially the velocity, and accurate joint force control are some of the main challenges of experimental implementation that will be discussed in the future works.

## 6. Conclusions

In this paper, we used a two-level controller, of MPC controller and whole-body controller, to realize generalized disturbance rejection for a biped model and get a better performance in push recovery, uneven ground walking and fast walking compared to a simplified model. The model can cover a 400 N external force, 4 cm uneven stairs and 10° slope without replanning the trajectory. Besides, this method can also control the model waking at a velocity of 6 km/h. The most important contribution of this paper is that we combine the simplified model and the whole-body model controller. Firstly, we use MPC to improve the ZMP tracking based on the simplified model. Secondly, the robot joint torque, joint acceleration and contact force are constructed as state variables, which facilitates the direct control of the included state variables. Thirdly, the task space controller is constructed, the mapping of the task space to the joint space control mode is realized through optimization, and whole-body motion is used to improve the walking stability. Finally, the ground friction constraint is added to solve the problem of a foot slipping and rotating in high-speed walking. The control framework used in this paper has good extensibility, in that it is easy to add or remove control targets to achieve more tasks.

## Figures and Tables

**Table 1:** Model parameters in simulation.

Description	Value
body mass and inertia	14 kg, [0.82,0.58,0.28] × 2 kg·m^2^
arm mass and inertia	3.10 × 2 kg, [0.006,0.133,0.133] × 2 kg·m^2^
hip mass and inertia	2.05 × 2 kg, [0.003.0.002,0.004] × 2 kg·m^2^
thigh mass and inertia	3.15 × 2 kg, [0.017,0.003,0.018] × 2 kg·m^2^
shank mass and inertia	3.15 × 2 kg, [0.002,0.037,0.036] × 2 kg·m^2^
ankle mass and inertia	0.94 × 2 kg, [0.002,0.001,0.002] × 2 kg·m^2^
foot mass and inertia	0.90 × 2 kg, [0.002,0.002,0.0008] × 2 kg·m^2^
$L_{arm}$	0.4 m
$L_{shoulder}$	0.504 m
$L_{body}$	0.459 m
$W_{hip}$	0.16 m
$L_{hip}$	0.056 m
$L_{thigh}$	0.274 m
$L_{shank}$	0.33 m
$L_{ankle}$	0.05 m
$H_{foot}$	0.061 m
$L_{foot}$	0.269 m
$W_{foot}$	0.16 m

**Table 2:** Weights and gains in simulation.

Task	Weight	Kp	Kd
$P_{com\to foot}$	1	[1000,1000,700]	[40,40,65]
$R_{b}$	0.6	[10,50,50]	[3.2,5.5,5.5]
$R_{fr}$	1	[150,150,90]	[33.9.33.9,24]
$R_{\mathrm{arm}}$	0.5	[50,50]	[5.5,5.5]
ZMP	0.003	[40,40]	[3.2,3.2]
F	0.001	-	-

## Supplementary Materials

The following are available online at https://www.mdpi.com/1424-8220/20/10/2971/s1, Video S1: Simulation environment; Video S2: Simulation of disturbance recovery based on MPC and whole-body dynamics control of biped walking.

## References

[1] Kajita S., Hirukawa H., Harada K., Yokoi K. (2014). Introduction to Humanoid Robotics.

[2] Vukobratovi M., Borovac B. (2004). Zero-moment point—Thirty five years of its life. Int. J. Hum. Robot..

[3] Ott C., Roa M.A., Hirzinger G. Posture and balance control for biped robots based on contact force optimization. Proceedings of the 2011 11th IEEE-RAS International Conference on Humanoid Robots.

[4] Kasaei M., Lau N., Pereira A. (2019). A Hierarchical Framework to Generate Robust Biped Locomotion Based on Divergent Component of Motion. arXiv.

[5] Kajita S., Morisawa M., Miura K., Nakaoka S.I., Harada K., Kaneko K., Yokoi K. Biped walking stabilization based on linear inverted pendulum tracking. Proceedings of the 2010 IEEE/RSJ International Conference on Intelligent Robots and Systems.

[6] Furusho J., Sano A. (1990). Sensor-based control of a nine-link biped. Int. J. Robot. Res..

[7] Nagasaka K.I. Stabilization of dynamic walk on a humanoid using torso position compliance control. Proceedings of the 17th Annual Conference on Robotics Society of Japan.

[8] Kagami S., Nishiwaki K., Sugihara T., Kuffner J.J., Inaba M., Inoue H. Design and implementation of software research platform for humanoid robotics: H6. Proceedings of the 2001 ICRA. IEEE International Conference on Robotics and Automation (Cat. No. 01CH37164).

[9] Hirai K., Hirose M., Haikawa Y., Takenaka T. The development of Honda humanoid robot. Proceedings of the 1998 IEEE International Conference on Robotics and Automation (Cat. No. 98CH36146).

[10] Yu Z., Zhou Q., Chen X., Li Q., Meng L., Zhang W., Huang Q. (2018). Disturbance rejection for biped walking using zero-moment point variation based on body acceleration. IEEE Trans. Ind. Inform..

[11] Li Z., Zhou C., Zhu Q., Xiong R., Caldwell D. (2015). Active Control of Under-actuated Foot Tilting for Humanoid Push Recovery. Proceedings of the 2015 IEEE/RSJ International Conference on Intelligent Robots and Systems (IROS).

[12] Vukobratovic M., Borovac B., Surla D., Stokic D. (2012). Biped Locomotion: Dynamics, Stability, Control and Application.

[13] Yoshino R. (2000). Stabilizing control of high-speed walking robot by walking pattern regulator. J. Robot. Soc. Jpn..

[14] Khatib O. (1987). A unified approach for motion and force control of robot manipulators: The operational space formulation. IEEE J. Robot. Autom..

[15] Hutter M., Hoepflinger M.A., Gehring C., Bloesch M., Remy C.D., Siegwart R. (2013). Hybrid operational space control for compliant legged systems. Robotics.

[16] Caron S., Kheddar A., Tempier O. Stair climbing stabilization of the HRP-4 humanoid robot using whole-body admittance control. Proceedings of the 2019 International Conference on Robotics and Automation (ICRA).

[17] Feng S., Whitman E., Xinjilefu X., Atkeson C.G. (2015). Optimization-based full body control for the darpa robotics challenge. J. Field Robot..

[18] Deng H., Ohtsuka T. A Parallel Code Generation Toolkit for Nonlinear Model Predictive Control. https://github.com/deng-haoyang/ParNMPC.

[19] MathWorks Simscape ™ Multibody ™ User’s Guide, © COPYRIGHT 2002–2019 by The MathWorks. https://ww2.mathworks.cn/products/simscape.html.

[20] Shafiee M., Romualdi G., Dafarra S., Chavez F.J.A., Pucci D. (2019). Online DCM Trajectory Generation for Push Recovery of Torque-Controlled Humanoid Robots. arXiv.

[21] Caron S. (2019). Biped Stabilization by Linear Feedback of the Variable-Height Inverted Pendulum Model. arXiv.

[22] Liu Y., Wensing P.M., Orin D.E., Zheng Y.F. (2015). Dynamic walking in a humanoid robot based on a 3D actuated Dual-SLIP model. Proceedings of the 2015 IEEE International Conference on Robotics and Automation (ICRA).

[23] Tedrake R., Kuindersma S., Deits R., Miura K. (2015). A closed-form solution for real-time ZMP gait generation and feedback stabilization. Proceedings of the 2015 IEEE-RAS 15th International Conference on Humanoid Robots (Humanoids).

